# The significance of chest ultrasound and chest X-ray in the diagnosis of children clinically suspected of pneumonia

**Published:** 2015

**Authors:** MB Rahmati, M Ahmadi, Malekmohamadi, S Hasanpur, SH Zare, M Jafari

**Affiliations:** * Department of Infectious Diseases, Children’s Clinical Research Development Center, Hormozgan University, School of Medical Sciences, Bandar Abbas, Iran,; ** Radiology Department, Hormozgan University of Medical Science, Iran,; *** Epidemiology Department, Hormozgan University of Medical Science, Iran,; **** Department of Pediatrics, Hormozgan University of Medical Sciences, Bandar Abbas, Iran

**Keywords:** pneumonia, chest X-ray, ultrasound

## Abstract

**Background.** Community-acquired pneumonia (CAP) is the most prevalent diseases and a significant determinant of morbidity and death global. This study intended to compare and evaluate the benefits and importance of chest X-ray and chest ultrasound in the investigation of CAP in children.

**Methods.** Study Population. One hundred children of one-month to five-years of age who suggested to the Children’s Hospital in Bandar Abbas for pneumonia were evaluated by chest ultrasound and chest X-ray by different radiologists.

**Results.** Evidence of involvement was recognized in 96% of the chest X-rays of those children, and also in 9% of the chest ultra-ultrasounds (6% opacity, 3% effusion). Pleural effusion was recognized in three of the children only by ultrasound.

**Conclusion.** The utilization of ultrasound is a suitable method to estimate the complication of pneumonia.

## Background

Pneumonia is the most usual infectious diseases of the lower respiratory tract in kids and a significant cause of morbidity and death global, affecting over 150 million children and leading to 3 million deaths of kids under 5 years old annually [**1**]. Community Acquired Pneumonia (CAP) is one common type of pneumonia. This disease is often accompanied by fever, cough, pleuritic chest pain, and dyspnea. If untreated, pneumonia can lead to respiratory failure, cardiac arrhythmia, and renal failure. The prevalence of this disease is of 12 in 1000 individuals [**2**]. Viral and bacterial pneumonias are usually connected including the virus of the higher respiratory region, lasting for several days with runny nose and cough. Viral pneumonia is typically associated with fever, which is milder than bacterial pneumonia. Tachypnea is usual clinical persistent symptom of pneumonia [**3**]. Difficulty in breathing, intercostal shrinkage, subcostal shrinkage, suprasternal retraction, and using accessory muscles of respiration are also common in pneumonia. Severe infections were associated with cyanosis, weakness, and respiratory fatigue especially in infants [**4**]. In patients with clinical signs of pneumonia, chest X-ray was extremely useful in the analysis of pneumonia. Symptoms of bacterial pneumonia are were usually the result of the direct invasion of bacteria of the chest cavity. These include pleural effusion and empyema. The correct diagnosis, appropriate and timely treatment, and identification of complications are considerably important. Diagnosis is based on clinical symptoms in greatest pieces of the universe. The analysis is verified with a chest X-ray. Ultrasound is a method used for the early analysis of pneumonia complications such as pleural effusion. In a recent study, ultrasound was used for the analysis of pneumonia. Will Bogus showed 93.4% sensitivity and 97.7% specificity of ultrasound in the analysis of pneumonia [**5**,**6**]. Therefore, we attempted to examine the patients with clinical signs of pneumonia and compare their chest X-ray and ultrasound outcomes to verify their analysis in order to reduce exposure to radiation, which can be problematic especially in children. In addition, we attempted to use ultrasound in suspicious cases and even diagnosed some symptoms in early stages.

## Methods

This proposed observational research was conducted on 100 patients aged from one-month to five-year children who were brought to Bandar Abbas Children’s Hospital and were hospitalized for pneumonia in 2012 and 2013. In addition to chest X-ray, on the third day, the children underwent an ultrasound evaluation. Clinical data along with age and sex from the patients’ records and the data from chest X-ray and ultrasound results were attached to the questionnaires. Criteria for inclusion in the research were: 

1- Age, which was between one-month to five-years.

2- Pneumonia symptoms such as fever, cough, rale lung sounds, respiratory distress symptoms (increased respiratory rate based on age: infants > 50 per minute, 1 to 5 years > 40 per minute, upper five years > 30 per minute) with chest X-ray results changes [**1**].

The exclusion criteria were the lack of cooperation of fathers and mothers or disinclination to play in the study after the explanation. The data was entered into the questionnaire, encoded, and entered into the statistical software SPSS version 19, and the t-test was applied to investigate the information.

## Results

In this current research, 100 patients were examined, which included 53 males, and 47 females. The middle years of subjects was 26.3 months. The difference between the mean age of males and females was significant (p-value = 0.019). Chest X-ray and ultrasound findings are displayed in **Table 1**, and the clinical data are given in **Table 2**.

**Table 1:** Ultrasound and chest X-Ray conclusions in children hospitalized for pneumonia

Chest X-ray	Chest Ultrasound	Pulmonary involvement
20	6	Consolidation
-	3	Effusion
31	-	Unilateral Reticular
45	-	Bilateral Reticular

**Table 2:** Clinical signs of the children hospitalized due to the diagnosis of pneumonia

Clinical Symptoms	% with the symptoms
Fever	80
Cough	97
Rhinorrhea, and nasal congestion	65
Conjunctivitis	5
Tachypnea	92
Retraction	64
Vomiting	16
Diarrhea	7
Otitis	2
Rale	58
Wheezing	26
Dyspnea	30
Nasal blade vibration	40
Cyanosis	13
Chest pain	6
Abdominal distension	1
History of hospitalization	15

## Discussion

The acute virus of the below airways (mainly pneumonia) is the principal reason of death in kids in developing countries. The prevalence of this infection is of 1.9 million children annually. The accurate diagnosis of pneumonia largely depends on clinical examination and precise imaging [**7**].

Confirming lung disease especially in children is considerably important. There is a balance between the high dosage of harmful potential rays in chest X-ray and the accurate diagnosis. Chest ultrasound is an alternative diagnostic method to evaluate pleura and pulmonary lesions and anterior mediastinum [**8**].

Ultrasound probably gives us a better image of the state of the lungs in the analysis of pneumonia because of the thinning of chest thoracic walls and large volume of the lungs in children compared to adults.

Chest X-ray requires cooperation to undergo a high dose of radiation and willingness to be transported to the radiology office. However, ultrasound was highly accessible and was conducted at the patients’ bedside in most cases, and since the results were instantly available, they were highly beneficial. Sadly, the ultrasound was uncertain due to its higher cost and inaccessibility of skilled ultrasound operators, and burden of transferring patients out of the hospital to another facility.

It was concluded that ultrasound was the main diagnostic device, because of its benefits mentioned above, and because it was performed at the patient’s bedside, along with the proper diagnosis of pulmonary infections. If the diagnostic procedures showed a high level of efficiency, they were used. As an outcome, the cases did not experience a high dosage of radiation; moreover, they were no longer required to be transferred outside the hospital.

Pulmonary lesions spread to the pleura up to 98.5% in adults, which can easily be recognized in ultrasound. However, children have smaller pulmonary tissue and there is a little chance for the lesion to spread to the pleura. Therefore, ultrasound is a suitable alternative to X-ray in the evaluation and consistency of children with pneumonia.

In this study, 100 children among 1 month and 5 years, who were hospitalized for pneumonia in Bandar Abbas hospital, were evaluated with chest X-ray and ultrasound. Evidence of involvement supporting the analysis of pneumonia was recognized in 96% of their chest X-rays while the findings supporting pneumonia were observed in 9% of the cases in chest ultrasound. The end was also consistent with X-ray results. Pleural flow was recognized in 3 cases who were diagnosed by ultrasound while no effusion was found in the X-ray results. This was similar to the other studies, which showed the significant of ultrasound in the analysis of pneumonia and extra pulmonary involvement.

In opposite to our findings, the findings in another researches confirmed the usefulness and high consistency between chest X-ray and ultrasound. Shebl showed that ultrasound could be applied for the analysis of pneumonia. They had 17 positive ultrasound criteria [**7**,**8**]. Also, there was no conclusion confirming pneumonia in chest X-ray results. Ressing showed that ultrasound acted 10 times stronger in the analysis of pneumonia in the German department compared to our study. The follow-up of patients was done by using ultrasound to analysis pneumonia. Finally, the results revealed that chest ultrasound had a high sensibility and specificity. Only 8% of the samples of pneumonia were not diagnosed by ultrasound while they were diagnosed by using chest X-ray [**9**]. Jean Eudes et al. showed that ultrasound was 9 times extra painful than chest X-ray in the analysis of pneumonia compared to our study. Jean Eudes et al. suggested that ultrasound is applied as the first diagnostic method compared to chest X-ray.

Since the subjects had a mean age of 26 months, younger infants had to be diagnosed with the ultrasound. The ultrasound device and probe specific to children that were used, differed from the device and probe used in similar studies. The ultrasound device applied in this research was the same as that used for adults. This device was also applied on children’s chest, which lowered the feeling of the system. Another reason for the difference between these results and similar studies was the restlessness and lack of cooperation of children in evaluating all thoracic directions by the sonographer. In similar studies, the ultrasound technique with a specific probe and the waves within the range of 7.5 to 10 MHZ were applied to examine all directions of the lungs (including parasagittal, transverse, coronal, mid-clavicle, anterior, posterior, mid-axillary chest lines) [**7**,**8**].

Community Acquired Pneumonia can affect interstitial and pleural pulmonary tissues. Based on similar studies, ultrasound had a high sensibility and specificity in the analysis of pleural effusion and peripheral lesions. However, only 9% of the positive ultrasound findings confirming pneumonia was observed in the current research because of the relationship of central and unilateral reticular zones (31%) bilateral reticular zones (45%) in chest X-ray. It should be remarked that there was no synchronization between chest X-ray and ultrasound in most cases. In this research, the patients were sent for an ultrasound on the third day. Before the ultrasound, patients were under antibiotic and other therapeutic measures. However, they would have been if the ultrasound had been performed at first. Then, X-rays would have been performed on the third day due to the risk of X-ray findings in sync with the clinical symptoms.

One of the controversial issues was that the reason for the insufficient detection in pneumonia patients through ultrasound was that they entered the study without considering the reason for their viral or bacterial infections, since turbidity was more likely to be recognized in the bacterial cases which counted for the majority of the samples in the intensive care units [**11**,**12**].

In this study, 97% cough, 96% tachypnea, 80% fever, and retraction were observed in 64% of the patients. These cases were consistent with those findings in other studies. This represented a major role of the clinical examination in the diagnosis of pneumonia, especially in developing countries. This also indicated the importance of the precise examination of the cases with cough, fever and tachypnea symptoms in early and immediate diagnosis [**8**].

## Conclusion

The current research indicated the lack of accuracy of ultrasound in the diagnosis of pneumonia and the detection of complications such as pleura.

**Recommendations**

Due to the small sample size as great as the inconsistent results with those in similar studies, it is suggested that more investigations with a larger sample size and evaluation of patients under 3 months old and thin chest walls (which shows usefulness of ultrasound) are conducted in order to confirm the application of ultrasound in the investigation of pneumonia. Also suggested that the subjects are divided into three groups including healthy subjects such as the ones in the control group, bacterial infections and viral infections, to compare and evaluate the ultrasound results.

**Limitations**

This study had several limitations. One was the reluctance of some parents to giving the consent for X-ray and removing their children from the hospital 3 days before the treatment, which led to the exclusion of several patients from the research. Another limitation was loss cooperation of the hospital staff in transferring patients to outside sources to conduct ultrasound by a radiologist, which prolonged the study to collect the patients.

**Acknowledgment**

This study is extracted from the thesis, At this moment, the authors appreciated to Children’s Clinical Research Development Center, Hormozgan University.
